# Comparative analysis of robotic vs. laparoscopic right hepatectomy: propensity score matching and machine learning analysis for outcome prediction

**DOI:** 10.3389/fsurg.2026.1739640

**Published:** 2026-03-27

**Authors:** Alessandro D. Mazzotta, Diab Samer, Giacomo Salina, Francesca Ratti, Federico Baldisseri, Paolo Magistri, Andrea Belli, Graziano Ceccarelli, Francesco Izzo, Marcello Giuseppe Spampinato, Nicola de’ Angelis, Patrick Pessaux, Tullio Piardi, Fabrizio Di Benedetto, Michele Ammendola, Luca Aldrighetti, Gianluca Mennini, Michele Tedeschi, Riccardo Memeo, Olivier Soubrane

**Affiliations:** 1 Department of General and Specialist Surgery, Sapienza University of Rome, Rome, Italy; 2 Department of Digestive, Oncological and Metabolic Surgery, Institut Mutualiste Montsouris, Paris, France; 3 Hepatobiliary Surgery Division, IRCCS San Raffaele Scientific Institute, Milano, Italy; 4 Hepatobiliary Surgery Division, Vita-Salute San Raffaele University, Milano, Italy; 5 Department of Computer, Control and Management Engineering, Sapienza University of Rome, Rome, Italy; 6 Unit of Hepato-Pancreato-Biliary Surgery and Liver Transplantation, University of Modena and Reggio Emilia, Modena, Italy; 7 Unit of Hepato-Biliary and Pancreatic Surgery, Istituto Nazionale Tumori IRCCS Fondazione G. Pascale, Napoli, Italy; 8 Unit of General Surgery, San Giovanni Battista Hospital, Foligno, Italy; 9 Unit of General Surgery, “Vito Fazzi” Hospital, Lecce, Italy; 10 Unit of Robotic and Minimally Invasive Digestive Surgery, Ferrara University Hospital, Ferrara, Italy; 11 Department of Visceral and Digestive Surgery, Unit of Hepato-Bilio-Pancreatic Surgery, Nouvel Hospital Civil, University Hospital of Strasbourg, Strasbourg, France; 12 Department of Hepatobiliary, Pancreatic and Digestive Oncological Surgery, Robert Debré University Hospital, Reims, France; 13 Science of Health Department, “Renato Dulbecco” Hospital, Digestive Surgery Unit, University “Magna Graecia” Medical School, Catanzaro, Italy; 14 Department of Hepato-Pancreatc-Biliary Surgery, “F. Miulli” General Regional Hospital, Bari, Italy; 15 Department of Medicine and Surgery, LUM University, Bari, Italy

**Keywords:** AI—artificial intelligence, laparoscopic surgery, liver, liver surgery, MIS, robotic surgery

## Abstract

**Background:**

Robotic right hepatectomy (RRH) is increasingly utilized as a minimally invasive liver surgery approach, yet its comparative effectiveness against laparoscopic right hepatectomy (LRH) remains debated. Complication prediction is crucial to efficiently choice the approach. This study aimed to compare RRH and LRH using a propensity score-matched (PSM) analysis and assess the predictive power of machine learning (ML), focusing on Random Forest-based feature selection.

**Methods:**

We retrospectively analyzed patients undergoing RRH and LRH, performing a 1:1 PSM based on preoperative characteristics. Postoperative outcomes were compared, and significant predictors of textbook outcomes (TO) were identified through univariate and multivariate analysis. A Random Forest model was developed, selecting key variables for TO achieving prediction.

**Results:**

After PSM, 30 RRH and 30 LRH patients were compared. RRH was associated with increased clamping (80% vs. 23.3%, *p* = 0.0001) and longer clamping duration (44 min vs. 35 min, *p* = 0.209) but resulted in a shorter hospital stay (6 vs. 7.5 days, *p* = 0.004) and fewer complications (13.3% vs. 53.3%, *p* = 0.001). Multivariate analysis identified Robotic surgical approach (OR 0.282, *p* = 0.004), Chronic hepatitis/Cirrhosis (OR 1.90, *p* = 0.034), and estimated blood loss (OR 2.001, *p* = 0.014) as significant predictors of TO. Feature selection via ML confirmed robotic surgical approach as one of the most relevant factors, alongside operative duration, estimated blood loss, and lesion size, in determining postoperative outcomes.

**Conclusion:**

The choice of surgical approach is a key determinant of outcomes, as demonstrated by its significance in both multivariate analysis and ML-based feature selection. RRH shows potential advantages in reducing hospital stay and complications while requiring more intraoperative management. Machine learning, particularly Random Forest, enhances outcome prediction by identifying critical surgical factors, supporting a more tailored approach in hepatobiliary surgery.

## Introduction

Liver resection represents one of the most complex procedures in abdominal surgery, mainly due to the intricate anatomy of the liver and its vascularization. Starting in the 1990s, laparoscopic liver resection (LLR) has been progressively adopted for the treatment of benign and malignant liver lesions, leading to an increasing global spread. The “Louisville Statement” of 2008 (1), followed by the recommendations of the Morioka consensus (2), confirmed its feasibility promoting a safe expansion of indications and an improvement in postoperative management. Despite these developments, LLR for major resections, such as right hepatectomy (RH), remains technically challenging for many surgeons. According to the Kawaguchi classification (3), right hepatectomy is considered a Class III procedure, reflecting its high technical complexity and the need for advanced surgical expertise. The intrinsic limitations of the laparoscopic techniques such as the fulcrum effect, two-dimensional vision, and reduced freedom of movement of the instruments—represent significant obstacles.

In this context, robotic liver surgery (RLS) has introduced a new dimension of precision, ergonomics, and visual control, thanks to systems equipped with magnified three-dimensional vision, instruments with seven degrees of freedom, and tremor filtering. Although recent years have seen an exponential increase in the number of studies comparing the outcomes of RLS and LLS in different types of hepatectomy and difficulty levels, high-level evidence is still missing (4–6). To evaluate the quality of the surgery single outcome measures may exhibit a low occurrence rate, hence constraining their efficacy as quality metrics (7).

Indeed, Textbook outcome (TO) is a novel composite measure that incorporates multiple favorable outcomes into a single metric and reflects the ideal postoperative course (8). Notably, TO has been described for multiple general surgery procedures, including colorectal, hepatobiliary, and esophageal surgeries (9–11).

In particular, no study to date has specifically investigated the achievement of TO in the context of minimally invasive right hepatectomy, despite its relevance as a composite measure of surgical quality.

Nevertheless, none of these has compared the intra and post operative aspects associated with these two surgical methods for right hepatectomy (RH). In addition, due to discrepancies in data and confounding variables in the included cohorts, it is difficult to draw reliable conclusions from these previous studies.

RLS has seen significant development since 2005, with an explosion of use especially in Asia and subsequently in Europe and the United States. Currently, RLS has been applied to a wide range of procedures: from minor and major resections to complex resections and living donor liver transplants. In 2023, the international consensus statements (12) sought to define the indications, advantages, and limitations of RLR, while emphasizing that the level of evidence often remains limited to case series, comparative analyses, and meta-analyses.

The purpose of this European multicenter retrospective study is to compare RH in robotic (RRH) and laparoscopic approach (LRH), through propensity score matching (PSM), and the application of machine learning algorithms (Random Forest) to identify the main predictors of clinical success, defined as TO.

## Patients and methods

### Study cohort

**Figure 1:**
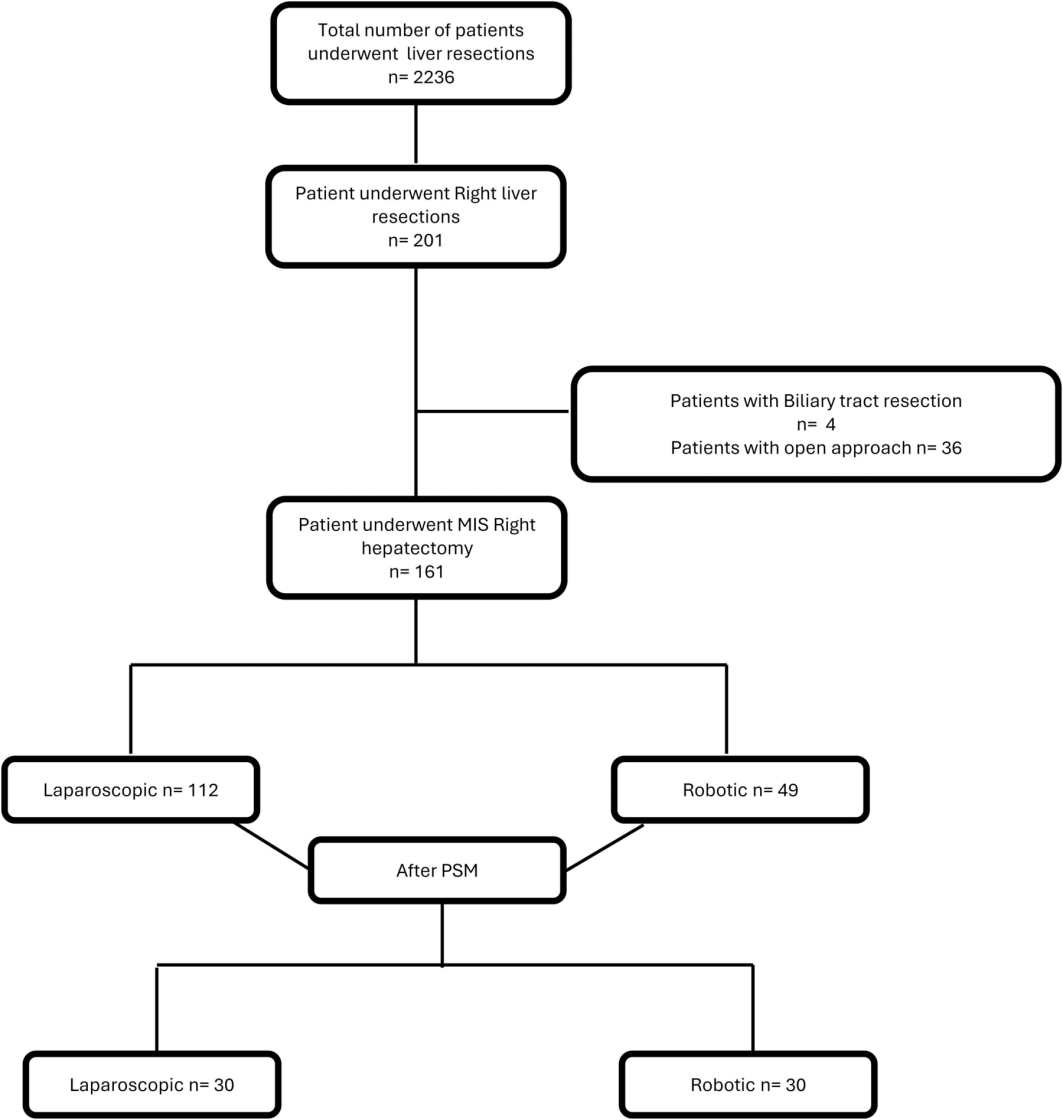
Flowchart of the patients before and after propensity score matching.

This is a retrospective, multicenter, observational study conducted across several European tertiary hepatobiliary centers. The study was approved by each participating institution's local ethics committee. Data were collected prospectively from institutional databases and retrospectively reviewed and anonymized for analysis. Between 1998 and 2022, a total of 2,236 patients underwent liver resections at participating centers. Among these, 201 patients underwent right liver resections. After excluding patients with biliary tract resection (*n* = 4) and open approach (*n* = 36), a final cohort of 161 patients who underwent minimally invasive right hepatectomy was included. Patients were divided based on surgical approach into two groups: laparoscopic (*n* = 112) and robotic (*n* = 49). A 1:1 propensity score matching (PSM) was performed, resulting in a final matched cohort of 60 patients (30 in each group). The patient selection process is depicted in Figure 1. Eligible patients were adults (≥18 years) who underwent minimally invasive right hepatectomy for benign or malignant liver lesions. Patients were excluded if they required biliary reconstruction, underwent an open approach, or had incomplete data regarding perioperative outcomes.

### Perioperative variables and definitions

The following variables were collected: demographics (age, sex, BMI), comorbidities (diabetes, cardiovascular disease, chronic hepatitis/cirrhosis), tumor characteristics (number, size, location), and surgical details [operative time, estimated blood loss, use of vascular clamping (Pringle Manouvre), conversion to open surgery]. In the robotic centers, inflow occlusion management was not standardized and clamping strategies (including the indication and duration of Pringle/clamping) varied across institutions and surgical teams.

Surgical complications were recorded and graded according to the Clavien-Dindo classification, with grade ≥IIIa defined as major complications (13).

Postoperative bile leak and post-hepatectomy liver failure were defined and graded according to the International study group of Liver Surgery (ISGLS) criteria. Specifically, bile leak was graded as A–C based on the impact on clinical management (grades B/C indicating clinically relevant bile leak) (14), and post-hepatectomy liver failure was graded as A–C according to deviation from the expected postoperative course (grades B/C indicating clinically relevant liver failure) (15).

Textbook Outcome (TO) was defined according to the International Delphi Consensus on Liver Surgery as the simultaneous achievement of all the following criteria (9):

- Absence of intraoperative incidents grade ≥2,
- Absence of postoperative bile leakage grade B/C,
- Absence of post-hepatectomy liver failure grade B/C,
- Absence of major postoperative complications (Clavien-Dindo ≥ IIIa) within 90 days,
- Absence of unplanned readmission related to major surgical complications within 90 days,
- Absence of 90-day or in-hospital mortality,
- R0 resection margin.

TO was treated as a **binary composite variable**. Failure to meet any single component resulted in non-achievement of TO (7).

Individual perioperative outcomes are reported separately from the composite TO, as length of stay and overall complications are not components of the TO definition.

### Statistical analysis

All quantitative data are expressed as median (interquartile range: IQR). Qualitative data are expressed as percentages. The Kruskal–Wallis test for continuous variables and Pearson's chi-square test were used when applicable, or Fisher's exact test for categorical variables. Pathological and perioperative variables were assessed as predictors of outcomes using Cox proportional hazards models. Statistical analyses were performed using SPSS 20.0 (SPSS Inc, Chicago, IL, USA). A *p*-value ≤ 0.05 was considered to indicate statistically significant differences.

### Propensity score matching

To reduce selection bias, a 1:1 PSM was performed using a nearest-neighbor algorithm without replacement. Variables included in the model were age, gender, ASA score, chronic hepatitis/cirrhosis, number of lesions, diagnosis type, and neoadjuvant therapy. Standardized mean differences <0.1 were considered indicative of successful matching. Patients were matched 1:1 by nearest-neighbor matching without replacement using the SPSS FUZZY extension. The matching tolerance was set to 0, such that matches were accepted only when propensity scores were identical (caliper = 0 on the propensity score scale). Covariate balance was evaluated using standardized mean differences (SMDs), with |SMD| < 0.10 indicating adequate balance; results are summarized in Love plots (Supplementary Figure S2). Missing data in the covariates used for propensity score estimation were minimal: one patient had a missing value for ASA class 3–4 and four patients had a missing value for neoadjuvant chemotherapy. Overall, five patients had at least one missing value among the PSM covariates; therefore, propensity scores were estimated using a complete-case approach (216/221 patients). No missing data were present for the PSM covariates in the post-matching cohort. Love plot are shown in Supplementary Figure S2.

In the post-PSM matched cohort, inflow occlusion (Pringle/clamping) was evaluated as a sensitivity analysis. Clamping use (yes/no) was compared using Fisher's exact test (two-sided), and clamping duration among clamped patients using the Mann–Whitney *U* test (two-sided). To assess whether the association between surgical approach and textbook outcome (TO) persisted after accounting for inflow occlusion, logistic regression models were fitted with TO as the dependent variable: Model 1 (TO∼approach) and Model 2 (TO∼approach + clamping yes/no). Adjustment for clamping duration was considered exploratory and performed as a complete-case analysis due to missing duration data in the LRH clamped subgroup.

### Machine learning analysis

The feature selection procedure employed in this study was designed to systematically identify the most relevant predictors of TO using a robust multi-method approach. Prior to feature selection, the data underwent comprehensive preprocessing. Categorical variables were imputed using the most frequent value and subsequently one-hot encoded to avoid multicollinearity, while numerical variables were imputed using the median and standardized to zero mean and unit variance. The machine learning models were trained using TO as a binary target variable derived strictly from the predefined composite criteria. No individual outcome (e.g., length of stay or overall complications) was used independently as a target variable.

Four distinct yet complementary feature selection methodologies were implemented to assess feature importance from different perspectives. First, univariate ANOVA *F*-test analysis was performed to evaluate each feature's individual predictive power through an *F*-test measuring linear dependency with the target variable. Second, recursive feature elimination (RFE) with logistic regression was applied, which iteratively removed the least important features while retraining the model, generating a feature ranking based on elimination order. Third, a Random Forest classifier was employed to compute Gini importance scores, quantifying each feature's contribution to reducing prediction error in an ensemble decision tree framework. Fourth, Pearson correlation analysis measured the linear association between each feature and the target variable.

To integrate these diverse perspectives, the results from each method were normalized to a common (0,1) scale and combined using a weighted scoring system. The final combined importance score for each feature was calculated as a weighted sum of the normalized scores (30% weight each to ANOVA, RFE inverse rank, and Random Forest importance, and 10% weight to absolute correlation). This aggregation approach was applied to ensure robustness against potential biases inherent in any single selection method. Features were then classified into three tiers based on their combined importance scores. High-importance features with scores exceeding 0.45 are indicated as primary predictors, while moderate-importance features (scores between 0.3 and 0.45) are to be conditionally included based on model performance requirements. Features scoring below 0.3 are to be generally excluded unless specific clinical relevance dictate otherwise.

## Results

### Preoperative characteristics

A total of 161 patients undergoing minimally invasive right hepatectomy were included, comprising 112 laparoscopic and 49 robotic procedures. After 1:1 propensity score matching, 60 patients were analyzed (30 per group). Before matching, patients in the robotic group exhibited a higher median BMI compared to the laparoscopic group [26.0 [25.0–27.1] vs. 24.91 [22.82–27.68], *p* = 0.020], and a significantly greater proportion underwent vascular clamping (83.7% vs. 25.1%, *p* < 0.001). The laparoscopic group had a significantly higher prevalence of underlying chronic hepatitis/cirrhosis (50.6% vs. 18.4%, *p* = 0.001), as well as more frequent neoadjuvant therapy (61.4% vs. 44.3%, *p* = 0.041) and a higher number of lesions [2 (1–4) vs. 1 (1–3), *p* = 0.014]. Following PSM, baseline variables were well balanced, with no significant differences in age, gender, ASA score, chronic hepatitis/cirrhosis, neoadjuvant therapy, or tumor burden between groups (Table 1).

**Table 1:** Preioperative characteristic Laparoscopic approach vs Robotic approach before and after the PSM.

				PSM 1:1		
Variable	Laparosocopic approach *n* = 112	Robotic approach *n* = 49	*P* value	Laparosocopic approach = 30	Robotic approach = 30	*P* value
Age	63 [56.12–71.92]	67 [36–74]	0.293	67.60 [36.86–72.12]	71 [57–74]	0.36
Gender (M)	101 (58.7)	30 (61.2)	0.753	19 (63.3)	17 (56.7)	0.753
BMI	24.91 [22.82–27.68]	26 [25–27.1]	0.020	24.91 [21.74–27.31]	26 [23.75–26]	0.231
ASA			0.018			0.223
1	25 (15.1)	2 (41)		4 (3.3)	1 (3.3)	
2	97(58.4)	37 (75.5)		19 (63.3)	24 (80)	
3	29 (17.6)	10 (20.4)		5 (16.7)	5 (16.7)	
Chronic hepatitis/cirrhosis	87 (50.6)	9 (18.4)	0.001	7 (23.5)	7 (23.3)	1
Neodjuvant Therapy	103 (61.4)	22 (44.3)	0.041	14 (46.7)	14 (46.7)	1
Number of lesions	2 [1–4]	1 [1–3]	0.014	2 [1–4]	2 [1–3]	0.352
Lesion size	40 [65–24.75]	38.5 [30–53]	0.600	35 [25–60]	40 [30–80]	0.390
Re-hepatectomy	48 (29.6)	3 (13)	0.096	10 (34.5)	2 (14.3)	0.166

[], IQR; (), %.

### Intraoperative outcomes

Clamping (pringle manouvre) was significantly more frequent in the robotic group both before (83.7% vs. 25.1%, *p* < 0.001) and after PSM (80.0% vs. 23.3%, *p* < 0.001). Clamping duration was notably longer in the robotic group before matching [54 (30–75) vs. 18.5 (3.5–38.25) minutes, *p* = 0.001], though the difference did not reach statistical significance after matching [44 [29.5–66.25] vs. 35 [7.5–40], *p* = 0.209]. Conversion to open surgery occurred in 7.6% of laparoscopic and 12.2% of robotic cases before matching (*p* = 0.302), and was rare after PSM (1 case in the laparoscopic group, none in the robotic group) (Table 2).

**Table 2:** Intraoperative and post operative results before and after the PSM.

				PSM 1:1		
Variable	Laparosocopic approach *n* = 112	Robotic approach *n* = 49	*P* value	Laparosocopic approach = 30	Robotic approach = 30	*P* value
Clamping	42 (25.1)	49 (83.7)	0.001	7 (23.3)	24 (80)	0.0001
Clamping duration	18.50 [3.50–38.25]	54 [30–75]	0.001	35 [7.5–40]	44 [29.50–66.25]	0.209
Operative time	270 [210–326.5]	300 [240–360]	0.177	240 [200–307]	300 [240–345]	0.215
Estimated blood loss	300 [125–500]	300 [200–400]	0.936	215 [100–375]	275 [200–400]	0.138
Lenght of stay	8 [6–12]	5 [4–7]	0.005	7.5 [6–11.75]	6 [4–6]	0.004
Conversion	13 (7.6)	6 (12.2)	0.302	1 (3.3)	0 (0)	0.313
General complication	105 (61)	11(22.4)	0.0001	16 (53.3)	4 (13.3)	0.001
Major complication	95 (20.3)	5 (10.2)	0.104	7 (23.3)	1 (3.3)	0.023
Re-intervention	10 (5.9)	0 (0)	0.084	3 (10)	0 (0)	0.076
Mortality	10 (3.9)	2 (4.2)	0.640	2 (6.7)	1 (3.4)	0.374
Trasfusion	19 (11.1)	3 (6.4)	0.341	2 (6.2)	2 (6.7)	/
TO (yes)	65 (37.8)	33(67.3)	0.0001	13 (43.3)	21 (70)	0.037
R1	17 (90)	4(8.7)	0.791	3 (10)	3 (10)	0.354
Re-admission	6 (3.5)	1(2)	0.0001	0 (0)	1 (3.3)	0.31

[], IQR; (), %; TO, yextbook outcome: defined as simultaneous fulfillment of: no intraop incident ≥2; no bile leak B/C; no liver failure B/C; no Clavien-Dindo ≥IIIa within 90 days; no 90-day readmission due to major complications; no 90-day/in-hospital mortality; R0 margin.

### Postoperative outcomes

The robotic group experienced significantly shorter hospital stays both before [5 (4–7) vs. 8 (6–12) days, *p* = 0.005] and after matching [6 (4–6) vs. 7.5 (6–11.75) days, *p* = 0.004]. Complication rates favored the robotic approach. General complications occurred in 61.0% of laparoscopic cases vs. 22.4% of robotic cases before matching (*p* < 0.001), and in 53.3% vs. 13.3% after matching (*p* = 0.001). Major complications were less frequent in the robotic group, significantly so after matching (3.3% vs. 23.3%, *p* = 0.023).

TO achievement was significantly higher in the robotic group, with a TO rate of 67.3% vs. 37.8% before matching (*p* < 0.001), and 70% vs. 43.3% after PSM (*p* = 0.037).

On multivariable analysis, the robotic approach was independently associated with improved TO (binary composite endpoint) (OR 0.282, 95% CI 0.115–0.690, *p* = 0.004), while chronic hepatitis/cirrhosis and increasing age were identified as negative predictors (Table 3).

**Table 3:** Uni- and multivariable analysis for failure to achieve textbook outcome (binary endpoint).

	Univariate			Multivariate		
Variable	OR	CI	*p*-value	OR	CI	*p*-value
Age (per 10y)	1.299	1.038–1.627	0.010	1.337	1.051–1.701	0.018
BMI	0.993	0.91–1.074	0.858			
Sex (M)	1.58	0.92–2.73	0.093			
ASA	1.157	0.98–1.36	0.081			
Chronic Hepatitis/Cirrhosis	2.23	1.290–3.881	0.004	1.94	1.051–3.612	0.034
Neoadjuvant therapy	2.205	0.70–2.07	0.499			
Robotic approach	0.295	0.158–0.547	0.001	0.272	0.112–0.663	0.004
Number of lesions	1.021	0.93–1.11	0.63			
Re-hepatectomy	1.579	0.81–3.076	0.180			
Clamping (min)	0.986	0.96–1.00	0.123			
Clamping (yes/no)	1.112	0.64–1.92	0.706			
Operative Time	1.002	0.999–1.005	0.161			
Estimated blood loss (per 100 ml)	1.116	1.00–1.237	0.008	1.128	1.004–1.267	0.043

OR, odds ratio; CI, confidence intervals.

In the contemporary-era sensitivity analysis (2018–2022), TO was achieved in 10/26 (38.5%) patients after LRH and in 33/49 (67.3%) patients after RRH (Fisher's exact test, two-sided *p* = 0.0265) (Supplementary Table S1).

In the post-PSM matched cohort, clamping remained significantly more frequent in RRH than in LRH (80.0% vs. 23.3%, *p* < 0.001; Supplementary Table S2), while comparisons of clamping duration were limited by missing time data in the LRH clamped subgroup (Supplementary Table S2). In stratified analyses, RRH showed a significantly higher TO achievement rate among clamped patients (70.8% vs. 14.3%, *p* = 0.012), whereas differences in major complications within strata were not statistically significant, likely due to very low event counts and limited subgroup sizes (Supplementary Table S3). Finally, sensitivity logistic regression analyses confirmed that the association between surgical approach and TO persisted after accounting for clamping: the RRH effect remained significant both before adjustment (OR 3.05, 95% CI 1.05–8.84; *p* = 0.0398) and after adjustment for clamping yes/no (OR 5.18, 95% CI 1.23–21.76; *p* = 0.0248) (Supplementary Table S4).

### Machine learning results and feauture selection

Notably, surgical approach emerged as the dominant predictor, followed by ASA score and chronic hepatitis/cirrhosis status (0.68). These results align well with clinical expectations, as surgical technique and patient physiological status are known to significantly influence postoperative outcomes. The strong performance of blood loss metrics further supports their established role as intraoperative indicators of surgical complexity and patient resilience. Interestingly, several variables traditionally considered important in clinical practice, such as maximum tumor size and operative duration, showed relatively modest importance scores (0.30–0.34). This suggests that while these factors may contribute to outcome prediction, their influence may be secondary to patient physiological status and surgical approach in our cohort (Supplementary Figure S1).

## Discussion

While many studies comparing RLR and LLR are emerging, there is still no robust evidence supporting the benefits of the robotic approach to recommend it as a first line in liver surgery. In the only available randomized trial (ROC’N’ROLL trial) (16) comparing RLR and LLR in patients with liver malignancy, there were no differences in postoperative quality of life, morbidity, and mortality between the two approaches. Moreover, in the latest ROBOT4HPB (12) consensus conference, experts and jury concluded that the robot may offer a benefit over laparoscopy in shortening the learning curve however, benefits for the patient are still to be determined. Nevertheless, many large series have speculated clear advantages for RLR.

In a series from the National cancer database, Duong et al. (5) compared 3,049 patients who underwent either RLR or LRL for stage I hepatocellular carcinoma and found that patients who underwent RLR had improved overall 1-year, 3-year, and 5-year survival rates; those results remained significant after PSM. In an another international multicenter study comparing RLR and LRL for major hepatectomies, RLR was found to be associated with fewer blood loss and lower rates of vascular clamping and conversion to open surgery. Furthermore, in a subset analysis of patients with cirrhosis, RLR was found to be associated with decreased morbidity and a shorter length of stay.

In our study, the overall complications were significantly lower in the robotic group (13.3% vs. 53.3% post-PSM, *p* = 0.001), as were the major complications according to Clavien-Dindo (3.3% vs. 23.3%, *p* = 0.023). It is hypothesized that the greater precision in surgical maneuvers, the stability of the image, and the quality of dissection offered by the robotic system may reduce the risk of bleeding, vascular or biliary injuries, and operative stress, resulting in a reduction of complications.

Despite RRH requiring a significantly more frequent use of vascular clamping (Pringle manouvre) in our case series (80% vs. 23.3% post-PSM, *p* < 0.001), no statistically significant differences in the duration of the procedure emerged between the two groups after matching. This result aligns with the conflicting evidence present in the literature. While some authors report a longer duration of RLR, an other study have shown that, with team experience, the operative time can be reduced to values comparable to laparoscopic ones and fall within the international benchmark standards (∼300 min for major hepatectomies) (17)..

This finding appears discordant with the largest available international comparative datasets, where robotic liver resection was associated with lower Pringle maneuver utilization compared with laparoscopy [e.g., 39.6% vs. 49.7% in a large PSM cohort reported by Sijberden et al. (18), and 47.1% vs. 63.0% in major hepatectomies reported by Liu et al.] (19).

Notably, procedure-specific series focused on right and extended right hepatectomy have reported no significant differences in Pringle use between approaches (42.3% vs. 49.8% after PSM in Chong et al.) (20), suggesting that the “direction” of clamping practice may be highly dependent on institutional strategy, case mix, and operative workflow. Residual imbalance in inflow occlusion persisted after matching, likely reflecting center-specific operative protocols and evolution of surgical strategy over time. We hypothesize that the higher clamping rate observed in our robotic group may reflect center-specific protocols aimed at optimizing parenchymal transection under a consistently dry operative field, particularly in a multicenter setting with heterogeneous transection techniques and anesthesiologic targets (e.g., low-CVP management). Moreover, given the long inclusion period and the more recent adoption of robotics in several centers, the robotic era may have coincided with a broader acceptance of intermittent inflow occlusion as a blood-sparing strategy.

Importantly, despite more frequent clamping, RRH in our series was not associated with longer operative time after matching and was coupled with improved postoperative outcomes, indicating that the clamping strategy likely represents a technical preference rather than a marker of inferior intraoperative control. However, we performed a sensitivity multivariable regression including both clamping use and clamping duration. The robotic approach remained independently associated with TO.

One of the key contributions of this study lies in the use of the TO as a composite endpoint and it incorporates multiple clinically relevant parameters——and is gaining recognition as a comprehensive measure of surgical quality. In our analysis, robotic surgery was significantly associated with TO achievement (OR 0.282, 95% CI 0.115–0.690, *p* = 0.004), reinforcing its potential impact on patient-centered outcomes.

Similar to our study, Sijberen et al. (18) used PSM to compare 3,010 patients undergoing RLR and LLR in an international retrospective cohort, and found RLR to be associated with a higher rate of TO, fewer blood loss, lower rates of vascular clamping, transfusion, and conversion, as well as lower overall morbidity and shorter operative time. In a subgroup analysis of major resections, TO tended to be higher in the RLR group but did not reach statistical significance. Similarly, in a recent meta-analysis with PSM comparing 9,355 patients undergoing RLR and LRL, RLR was associated with better surgical outcomes, fewer blood loss, a lower rate of conversion and a higher rate of R0 resection. In the subgroup analysis of major hepatectomies, only blood loss and conversion rate remained significantly lower in the robotic group. Length of stay, although improved in the robotic group, was analyzed separately and was not included in the TO definition, in accordance with the international consensus. We showed that RRH was associated with a significantly higher rate of TO compared with the laparoscopic approach, both before and after propensity score matching. Moreover, RLR showed lower rates of overall and major complications as well as a shorter length of stay, while maintaining a comparable safety profile in terms of operative time and conversion rate. We performed additional sensitivity analyses in the post-PSM matched cohort to address the residual imbalance in inflow occlusion strategy, which likely reflects center-/team-specific intraoperative clamping policies. The unadjusted model showed a significant association between approach and TO (RRH vs. LRH: OR 3.05, 95% CI 1.05–8.84; *p* = 0.0398). After adjustment for clamping (yes/no), the approach effect remained significant (OR 5.18, 95% CI 1.23–21.76; *p* = 0.0248). Inclusion of clamping time was limited by substantial missing duration data in the LRH clamped subgroup; therefore, clamping-time adjustment was considered exploratory and is reported as a complete-case sensitivity analysis.

The feature selection process validated the clinical significance of multiple established predictors of surgical success, such as surgical approach, ASA score, and chronic hepatitis/cirrhosis. The integration of ML-based ranking with clinical experience enhances the reliability of ML tools for validating and prioritizing established risk factors in minimally invasive liver surgery.

Variables traditionally deemed crucial, such as tumor size and operative duration, exhibited only moderate significance in our analysis. This indicates that these parameters, although not insignificant, may have a subordinate effect relative to patient-specific physiological status and surgical strategy. This underscores the potential advantages of integrated or composite indices, such as intraoperative blood loss and TO, in effectively capturing the complexity of surgical risk. In contrast to classical regression models, ML algorithms are capable of modeling non-linear interactions, addressing multicollinearity, and uncovering concealed data structures.

Our study presents several limitations related to its retrospective and observational design. Initially, while propensity score matching was utilized to reduce selection bias, residual confounding variables—such as differences in surgical technique, perioperative treatment, and institutional protocols—may have impacted the outcomes. Secondly, the study cohort, although being sourced from several high-volume institutions, encompassed a restricted number of robotic right hepatectomies, potentially impacting the generalizability of the results. Furthermore, the results encompass an extended inclusion period (1998–2022), during which surgical technologies, perioperative care, and patient selection criteria have presumably advanced over time and robotic programs were implemented later than laparoscopy across centers. Although a contemporary-era sensitivity analysis (2018–2022) was performed, residual confounding related to temporal trends cannot be completely excluded. Another significant limitation of this study is that the use of Pringle/clamping (indication and duration) represents a non-standardized intraoperative choice, influenced by the complexity of the case and, above all, by the policies and preferences of individual teams/centers (particularly in robotic centers). Consequently, despite having conducted sensitivity and stratified analyses, it is not possible to completely exclude residual confounding, and the estimates are limited by the small number of some subgroups and by incomplete data on the duration of clamping in the laparoscopic group.

A further issue is the lack of consistent reporting for intraoperative variables, including blood loss, dissection time, and console utilization, which could have improved the assessment of operating efficiency. Furthermore, the experience of surgeons and their learning curves, which are recognized to influence outcomes in both laparoscopic and robotic procedures, were not systematically evaluated. Ultimately, although ML research was employed to ascertain determinants of surgical success, it is still exploratory and should be taken cautiously due to the risk of overfitting on a limited dataset. Despite the methodological rigor, the exploratory nature of ML applications in surgical datasets should be emphasized. The relatively small sample size, particularly in the robotic subgroup, poses a risk of overfitting. External validation in larger, prospective cohorts is necessary to confirm the stability and applicability of the identified predictors. Following studies should seek to corroborate these results using prospective, randomized controlled trials employing standardized surgical methods and inclusion criteria. The identification of a limited set of highly informative variables establishes a basis for the development of clinical decision support systems (CDSS). These may aid in preoperative risk stratification, patient selection, and surgical planning by providing real-time, individualized predictions of surgical outcomes. Integrating these models into digital health platforms may improve clinical decision-making in hepatobiliary surgery. The incorporation of artificial intelligence—encompassing predictive analytics, image-guided navigation, and intraoperative decision support—could significantly improve the safety and effectiveness of robotic liver surgery. Specifically, real-time data acquisition and federated learning models among institutions could facilitate dynamic benchmarking and personalized risk classification. Ultimately, cost-effectiveness studies and long-term oncological results must be integrated to ascertain the true benefit of the robotic technique in hepatobiliary surgery.

## Conclusion

This European multicenter study demonstrates that robotic right hepatic resection is associated with a significantly higher rate of TO compared to the laparoscopic counterpart, even after propensity score matching. Machine learning analysis to identify the main predictors of surgical success. The multi-method feature selection procedure highlighted how the surgical approach, the physiological status of the patient (ASA score) and the presence of chronic hepatitis/cirrhosis are the main determinants of the achievement of the TO, variables traditionally considered central, such as tumor size and operative duration, showed a lower predictive impact.

## Data Availability

The datasets generated and/or analyzed during the current study are available from the author on reasonable request.
