# Supplementary material for: Comparative analysis of robotic vs. laparoscopic right hepatectomy: propensity score matching and machine learning analysis for outcome prediction
Source: Front Surg. 2026 Mar 27;13:1739640. doi: 10.3389/fsurg.2026.1739640 (PMC13065511; doi:10.3389/fsurg.2026.1739640)
Supplement: Supplementary file 1 [file Supplementaryfile1.docx]

Supplementary Table 1. Textbook outcome (TO) by surgical approach in the contemporary era (2018–2022).

| **Surgical approach** | **TO achieved, n (%)** | **TO not achieved, n (%)** | **Total, n** | **p value*** | |
| --- | --- | --- | --- | --- | --- |
| Laparoscopic right hepatectomy (LRH) | 10 (38.5) | 16 (61.5) | 26 | 0.0265 |  |
| Robotic right hepatectomy (RRH) | 33 (67.3) | 16 (32.7) | 49 |  |  |
| Overall | 43 (57.3) | 32 (42.7) | 75 |  |  |

Supplementary Table 2. Inflow occlusion (Pringle/clamping) in the post-PSM matched cohort.

| **Variable** | **RRH (n=30)** | **LRH (n=30)** | **p value** |
| --- | --- | --- | --- |
| Clamping, n (%) | 24 (80.0) | 7 (23.3) | **<0.001** |
| Clamping time (min),  median (IQR)‡ | 48 (34.5–67.5) (n=23) | 40 (35–40) (n=3) | 0.295§ |

Supplementary Table 3. Stratified comparison of RRH vs LRH in the post-PSM matched cohort according to clamping status.

|  | **Outcome** | **RRH, n/N (%)** | **LRH, n/N (%)** | **p value*** |
| --- | --- | --- | --- | --- |
| **Clamping: Yes** | Textbook outcome (TO) achieved | 17/24 (70.8) | 1/7 (14.3) | **0.0124** |
|  | Major complications | 0/24 (0.0) | 1/7 (14.3) | 0.2258 |
| **Clamping: No** | Textbook outcome (TO) achieved | 4/6 (66.7) | 12/23 (52.2) | 0.6628 |
|  | Major complications | 1/6 (16.7) | 6/23 (26.1) | 1.000 |

**Supplementary Table 4.** Sensitivity analyses: effect of surgical approach on textbook outcome (TO) in the post-PSM matched cohort (n=60), before and after adjustment for inflow occlusion.

|  | **Covariates** | **RRH vs LRH effect (OR, 95% CI)** | **P value** |
| --- | --- | --- | --- |
| Model 1 (unadjusted) | Approach only | 3.05 (1.05–8.84) | 0.0398 |
| Model 2 (adjusted) | Approach + Clamping (yes/no) | 5.18 (1.23–21.76) | 0.0248 |
| Model 3 (exploratory complete-case)† | Approach + Clamping + Clamping time | 2.44 (0.52–11.30) | 0.256 |
