# Supplementary figures and images for: Comparative analysis of robotic vs. laparoscopic right hepatectomy: propensity score matching and machine learning analysis for outcome prediction
Source: Front Surg. 2026 Mar 27;13:1739640. doi: 10.3389/fsurg.2026.1739640 (PMC13065511; doi:10.3389/fsurg.2026.1739640)

Supplementary Figure 1.

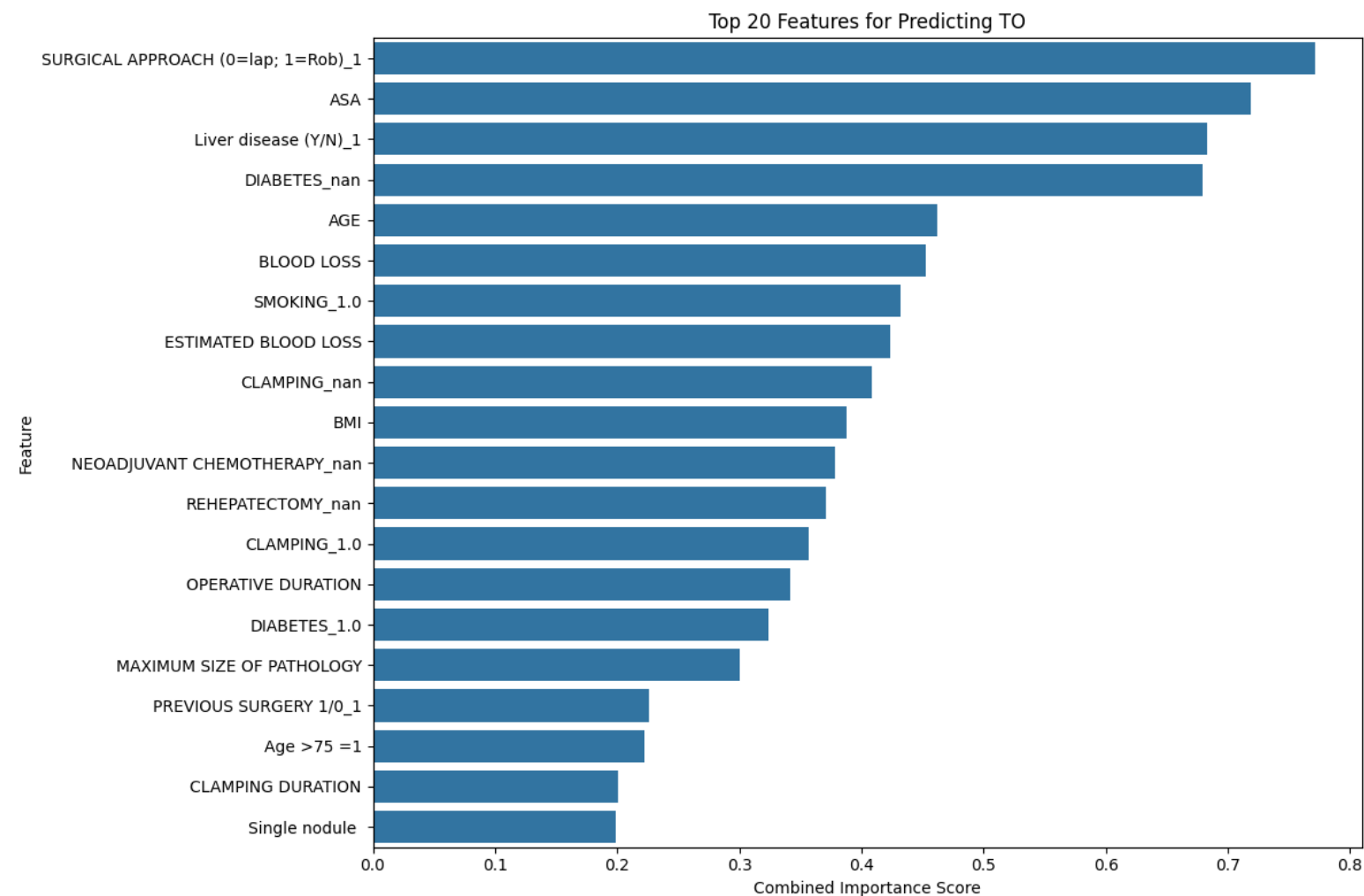

Supplementary Figure 2.

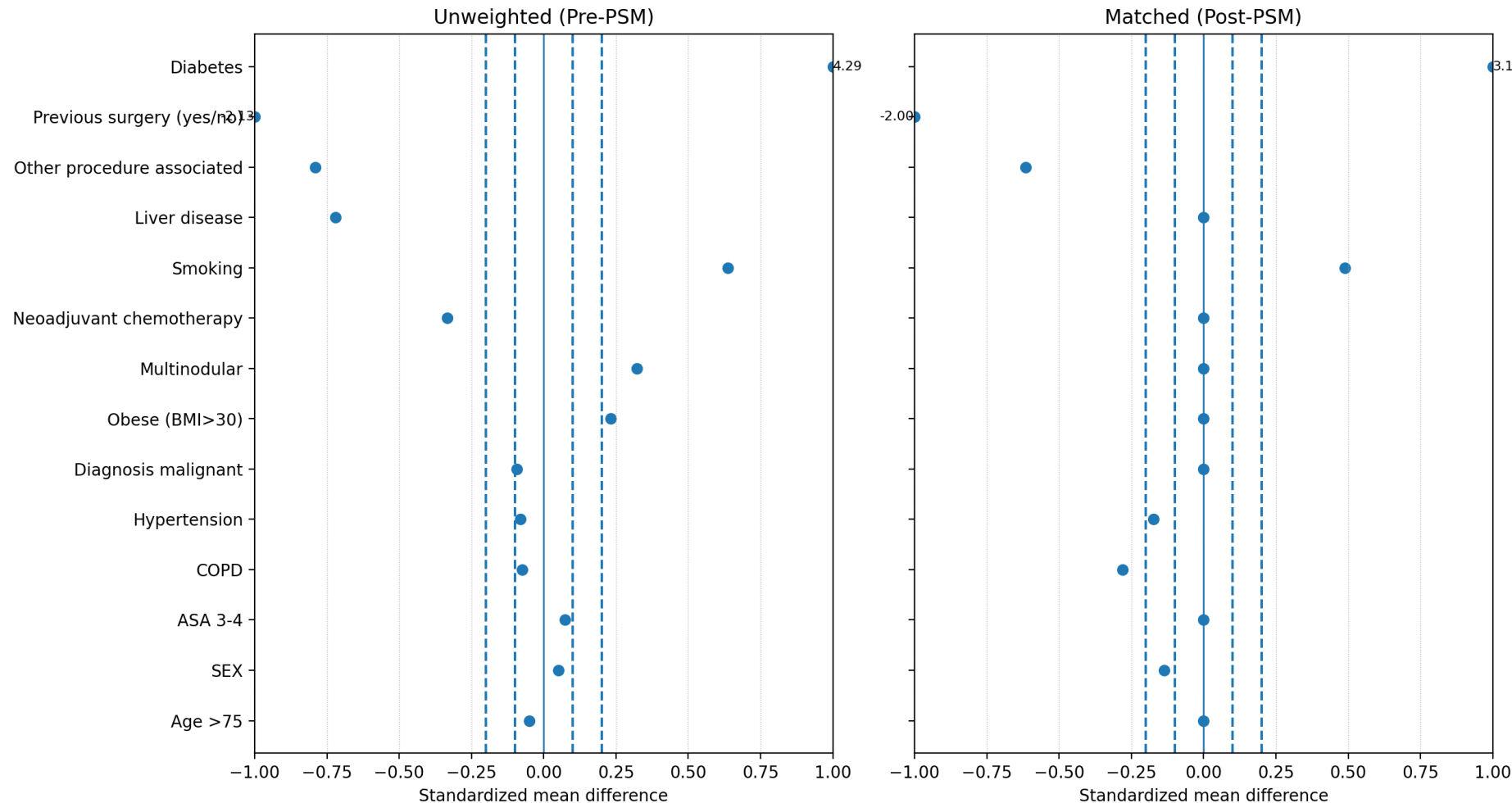

Supplement: Supplementary file 2 [file Supplementaryfile2.pdf]
